# Evolutionary genomics of *Entamoeba*

**DOI:** 10.1016/j.resmic.2011.01.007

**Published:** 2011-07

**Authors:** Gareth D. Weedall, Neil Hall

**Affiliations:** Institute of Integrative Biology, University of Liverpool, Crown Street, Liverpool L69 7ZB, UK

**Keywords:** *Entamoeba*, Genetic diversity, Genomics, Molecular epidemiology

## Abstract

*Entamoeba histolytica* is a human pathogen that causes amoebic dysentery and leads to significant morbidity and mortality worldwide. Understanding the genome and evolution of the parasite will help explain how, when and why it causes disease. Here we review current knowledge about the evolutionary genomics of *Entamoeba*: how differences between the genomes of different species may help explain different phenotypes, and how variation among *E. histolytica* parasites reveals patterns of population structure. The imminent expansion of the amount genome data will greatly improve our knowledge of the genus and of pathogenic species within it.

## 1 Introduction

*Entamoeba histolytica* is a parasite of the human large intestine, commonly contracted by ingesting contaminated water or food. The parasite has a two-stage life cycle in which the infective stage in the environment is the cyst and the motile stage within the host is the trophozoite. Infection with the parasite is endemic in many parts of the world where sanitation infrastructure is poor; in other places infection tends to be restricted to certain groups, such as residents in institutions for the mentally handicapped and men who have sex with men (Haghighi et al., 2002, 2003; Rivera et al., 2006). The global prevalence of infection was estimated in 1986 to be 10% of the world’s population (Walsh, 1986). Of these, 90% were estimated to be asymptomatic carriers and 10% to develop symptoms of invasive amoebiasis. Amoebiasis results from invasion of the gut wall, leading to diarrhoea and dysentery (bloody stools), and in some cases to colonisation of organs (commonly the liver) and production of abscesses. The global prevalence estimate was made prior to the re-description of *E. histolytica* in 1993 that separated it into two species (Diamond and Clark, 1993): the potentially virulent *E. histolytica* and the avirulent *Entamoeba dispar*. Despite this change, invasive amoebiasis still appears to be a relatively rare outcome of *E. histolytica* infection.

Understanding what determines the outcome of infection, and the nature of amoebic virulence more generally, motivates a substantial body of *Entamoeba* research. As part of this effort, the genome sequences of a number of *Entamoeba* species have been determined. These offer a fascinating insight into the evolution of these organisms. Here we review a number of notable features of *Entamoeba* genomes, from the point of view both of the evolution of different species lineages and of genetic diversity among *E. histolytica* populations.

## 2 Whole-genome sequences of *Entamoeba* species

**Fig. 1:**
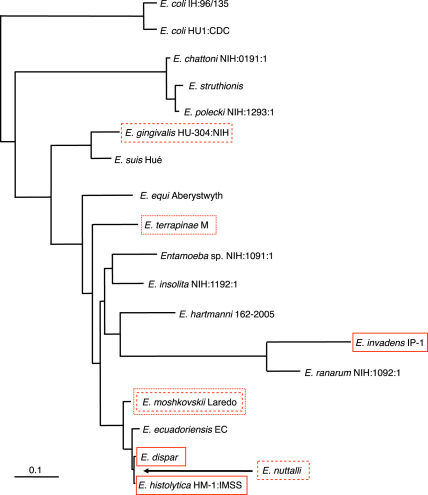
Phylogeny of *Entamoeba*, adapted from Clark et al. (2006b). Phylogeny based on small subunit rRNA genes. Red boxes indicate species for which genomes have been sequenced. Red dotted boxes indicate species for which there is low coverage shotgun sequence data. Red dashed boxes indicate species due to be sequenced.

The *Entamoeba* genus contains many species infecting a wide range of hosts. The simplest morphological feature used to distinguish species is the number of nuclei in the cysts, commonly 1, 4 or 8, although some species like the oral parasite *Entamoeba gingivalis* do not form cysts. The phylogeny of the genus shows often large evolutionary distances between *Entamoeba* species. Fig. 1 shows a phylogeny of the *Entamoeba* genus, and indicates species for which genome sequence data are, or are soon to be, available.

Table 1 shows details of the *Entamoeba* genomes sequenced and assembled so far. The most up-to-date annotations and many genome-level datasets are presented at the amoebaDB website (Aurrecoechea et al., 2011; www.amoebadb.org).

The draft genome sequence of *E. histolytica* (strain HM1:IMSS) was published and analysed in 2005 (Loftus et al., 2005) and was subsequently re-assembled and re-annotated (Lorenzi et al., 2010; Clark et al., 2007). The genome assembly consists of 20,800,560 base pairs of DNA in 1496 scaffolds. The genome is very AT-rich (approximately 75% AT) and quite gene-rich: around half of all assembled sequence is predicted to be coding sequence, with 8333 annotated genes.

*E. dispar* is the closest described relative of *E. histolytica* and is morphologically identical, being designated a separate species in 1993 (Diamond and Clark, 1993). It is not known to be virulent, but rather to live as a commensal in the gut. The genome assembly is of a similar size to that of *E. histolytica*, consisting of 22,955,291 bp of DNA in 3312 scaffolds. Its AT content is also similar to *E. histolytica* (approximately 76.5% AT) and a similar proportion is predicted to be coding sequence, with 8749 annotated genes.

*Entamoeba invadens* is a parasite of reptiles and, although only distantly related to *E. histolytica*, is an important model for the encystation process, since it can be induced to encyst in axenic laboratory culture, while *E. histolytica* cannot. The genome appears to be larger than that of *E. histolytica* or *E. dispar*, consisting of 40,888,805 base pairs of DNA in 1149 scaffolds. It is also slightly less AT-rich (approximately 70% AT). Approximately 38% of all the sequence is predicted to be a coding sequence, with 11,549 annotated genes. In addition to these genome assemblies, low coverage shotgun sequencing projects have been carried out on the genomes of a further two *Entamoeba* species: *E. terrapinae* and *Entamoeba moshkovskii*. Sequencing reads are publicly available.

Next-generation sequencing platforms allow rapid sequencing of entire genomes. *E. moshkovskii*, a putative free-living species which has nonetheless been shown to infect humans (Ali et al., 2003), has been sequenced by our group (manuscript in preparation). The oral parasite *E. gingivalis*, which has not been shown to form cysts, will also be sequenced. *Entamoeba nuttali*, a pathogen of monkeys and apparently more closely related to *E. histolytica* than *E. dispar* (Tachibana et al., 2007), will be sequenced by colleagues at the J. Craig Venter Institute (Dr. Elisabet Caler, personal communication). In addition to these species, our group and colleagues at the JCVI are in the process of sequencing multiple strains of *E. histolytica* to assay intraspecies genomic diversity. All of these data will be made publicly available. As the number of sequenced genomes increases, our understanding of the evolutionary processes shaping these genomes will improve.

## 3 Structure and organization of the genome

The content of the *E. histolytica* genome has been reviewed extensively elsewhere (Clark et al., 2007). A number of interesting evolutionary features of the genome have been highlighted, not least the significant number of genes (at least 68) that appear to have been gained by horizontal gene transfer from bacteria (Loftus et al., 2005; Clark et al., 2007; Alsmark et al., 2009). These genes tend to be involved in metabolic processes characteristic of the anaerobic lifestyle of the organisms (Rosenthal et al., 1997; Field et al., 2000; Andersson et al., 2006), and the majority of transfers appear to have been ancient, as orthologues are found in both *E. histolytica* and the highly divergent *E. invadens* (Roy et al., 2006).

Much remains unknown about the large-scale structure and architecture of *Entamoeba* genomes. For instance, neither the natural ploidy nor the haploid number of chromosomes is known, although there are estimates of both. Hybridisation of gene markers to pulsed-field gels identified linkage groups and a haploid number of 14 chromosomes (Willhoeft and Tannich, 1999). Suggestions of tetraploidy and diploidy have been advanced: tetraploidy based on labelling patterns in PFGE (Willhoeft and Tannich, 1999) and diploidy based on fluorescence in situ hybridisation to putative single-copy genes (Ghosh et al., 2000). Ploidy appears to be variable even within a cell lineage under different growth conditions (Mukherjee et al., 2008), although whether this results from growth in laboratory culture only or also occurs in nature is not known.

Studies of the molecular karyotype of *E. histolytica* show complex patterns of differences in chromosome sizes between strains and a mixture of linear and circular DNA molecules (Willhoeft and Tannich, 1999; Riveron et al., 2000). A number of circular DNA structures occur in *E. histolytica* (Dhar et al., 1995; Lioutas et al., 1995; Báez-Camargo et al., 1996; Riveron et al., 2000). The rRNA genes occur on one such molecule that exists in multiple copies per nucleus (Bhattacharya et al., 1998). Lorenzi et al. (2010) suggested that segmental duplications detected in the genome assembly might, in fact, represent some of these circular molecules (Lorenzi et al., 2010). These structures could be important for parasite phenotypes and their presence raises many intriguing questions. For instance, is their copy number different from that of the ‘core’ chromosomes and do they segregate in the same way?

**Fig. 2:**
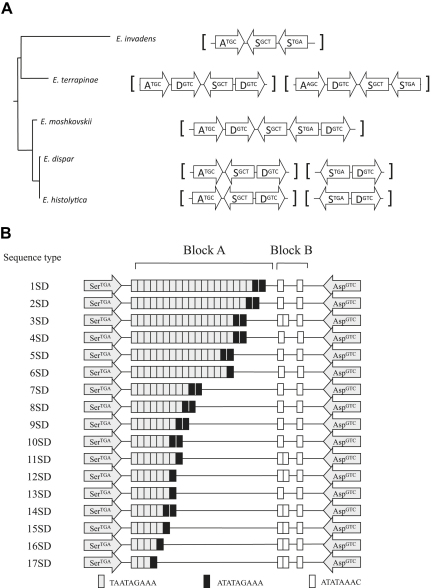
Diversity of tRNA gene arrays, from Tawari et al. (2008). (A) Rearrangements of tRNA gene array units containing alanine, serine and aspartic acid tRNA genes, among *Entamoeba* species. Genes are represented by arrows containing single-letter amino acid codes (A = alanine, S = serine, D = aspartic acid) and superscripted anticodons. (B) Schematic representation of polymorphism in the intergenic DNA between SD array units, comprising serine (Ser^TGA^) and aspartic acid (Asp^GCT^), among *E. histolytica* isolates.

An unusual feature of the genomic organisation of *E. histolytica* is that its tRNA genes occur in arrays of tandemly duplicated combinations of genes separated by DNA that may contain short tandem repeats (Clark et al., 2006a; Tawari et al., 2008). It has been suggested that these could act as telomeres, as a telomeric sequence has not been identified in *Entamoeba* genomes. The arrangement of tRNA genes appears to be quite variable and to have changed in the evolution of different species lineages. Fig. 2 shows examples of different arrangements of tRNA genes in different species. The DNA between the tRNA genes in a number of arrays is highly variable between isolates of *E. histolytica* (and *E. dispar*) and they have been developed for use as population genetic markers (Ali et al., 2005). However, the pattern of variable short tandem repeats seen in *E. histolytica* is not seen in *E. moshkovskii*, where these regions are variable but not repetitive (Tawari et al., 2008).

## 4 Transposable elements, synteny and genomic rearrangements

In parasitic protozoa such as *Plasmodium*, large-scale genome architecture appears to be quite stable over large evolutionary distances. Distant relatives in the genus (the human parasite *Plasmodium falciparum* and the rodent parasites *Plasmodium yoelii*, *Plasmodium chabaudi* and *Plasmodium berghei*) generally possess a shared ‘core genome’ covering the central region of each chromosome, while the subtelomeric chromosomal regions tend to be more variable and species-specific and contain members of large gene families (Kooij et al., 2005). A similar distribution of variability is seen among different *P. falciparum* isolates (Volkman et al., 2007).

Such a pattern cannot be seen in *Entamoeba*, as the karyotype is not yet well enough resolved and its complexity suggests a degree of genomic plasticity within *E. histolytica*. Such variability of chromosome size has been described in other protozoa (Adam, 1992; Blaineau et al., 1991; Melville et al., 1999) and genomic plasticity and instability may be an important feature of the evolution of *Entamoeba*, both within and between species. Genome rearrangement associated with invasive disease has been suggested as one possible explanation for the different tRNA-STR genotypes detected in liver abscess and stool-derived parasites from the same infected person (Ali et al., 2007).

**Fig. 3:**
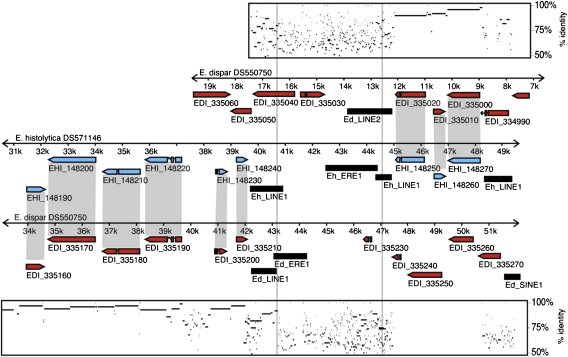
Example of genome rearrangement between *E. histolytica* and *E. dispar* associated with the presence of transposons, from Lorenzi et al. (2008). Synteny (represented by grey bars connecting orthologous *E. histolytica* and *E. dispar* genes) switches to a different *E. dispar* scaffold across the region containing LINE, SINE and ERE elements. This is also shown by percentage sequence identity plots which show that scaffolds lose significant sequence identity between species across the LINE, SINE and ERE elements.

Transposons and repetitive DNA, which are present in abundance in *Entamoeba*, may facilitate genome rearrangements. A comprehensive study of the repetitive elements of three *Entamoeba* genomes (Lorenzi et al., 2008) found hundreds of copies of LINE and SINE elements, as well as *Entamoeba*-specific repeats. These *Entamoeba*-specific ERE1 and ERE2 sequences represent a large proportion of the genome of *E. histolytica*. The ERE2 sequence may be unique to *E. histolytica*, as it was found in neither *E. dispar* nor *E. invadens* (Lorenzi et al., 2008). LINE and SINE elements are class I transposons (propogated via reverse transcription). Class II transposons (DNA transposons) have also been detected in *Entamoeba* species, and though they are rare in *E. histolytica* or *E. dispar*, they appear to be much more prevalent in *E. invadens* and *E. moshkovskii* (Pritham et al., 2005). These results suggest expansion and contraction of the number of transposable elements in different lineages, with likely consequences for genome rearrangement. Fig. 3 shows an example of a break in synteny between *E. histolytica* and *E. dispar* across a chromosome region containing repetitive elements.

Comparisons of the genomes of *E. histolytica* and *E. dispar* show that transposons have been active since these species diverged (Willhoeft et al., 2002; Shire and Ackers, 2007; Lorenzi et al., 2008). Active transposition may still be occurring. Huntley et al. (2010) identified a number of putative recent transpositions of *Eh*SINE1 elements in the HM1:IMSS genome (Huntley et al., 2010).

## 5 Gene families and diversity

**Fig. 4:**
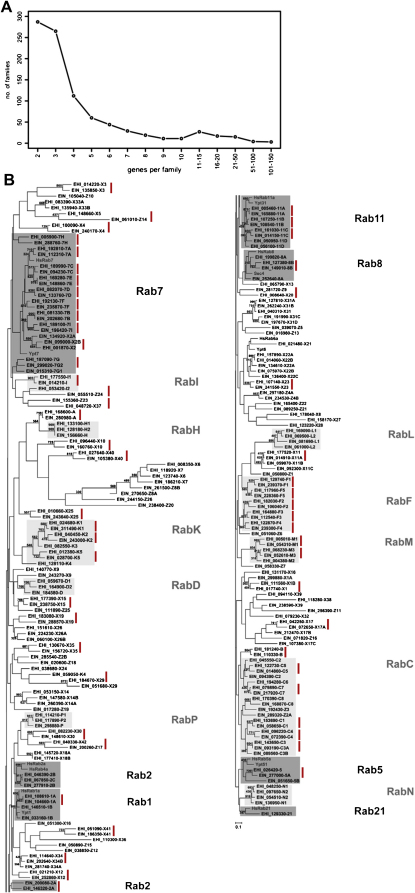
Large gene families in *Entamoeba*. (A) Size distribution of *E. histolytica* multi-gene families, showing a number of very large families, from Lorenzi et al. (2010). (B) Phylogeny of Rab GTPase genes in *E. histolytica* and *E. invadens*, adapted from Nakada-Tsukui et al. (2010). Putative orthologous gene pairs are indicated with red bars, and their frequency indicates an ancient radiation of Rab GTPase genes.

Possession of large gene families often indicates the importance and complexity of particular processes. *E. histolytica* contains a number of large multi-gene families (Lorenzi et al., 2010; see (Fig. 4a)). A large gene family encodes a group of AIG1-like GTPases (Lorenzi et al., 2010). Their precise function is unknown, but differential expression suggests they may be associated with virulence and/or adaptation to the intestinal environment (Davis et al., 2007; Biller et al., 2010; Gilchrist et al., 2006). Members of the AIG1-like family, among a number of gene families, often occur near to transposons (Lorenzi et al., 2010). It remains to be seen whether gene duplication and subsequent growth of gene families is promoted by the proximity of these elements. Another large gene family encodes proteins homologous to a bacterial fibronectin-binding protein (BspA of *Bacteroides forsythus*), which encodes a large number (75–116) of proteins containing leucine-rich repeats (Davis et al., 2006; Lorenzi et al., 2010). At least one member of this family is expressed at the parasite surface (Davis et al., 2006). A survey of *E. invadens* sequence reads indicated the presence of multiple copies of these leucine-rich repeat-containing genes (Wang et al., 2003). Differential gene expression within gene families occurs, although it is unclear whether gene expression is controlled so that single gene family members are expressed at any one time, as seen in important *Trypanosoma* and *Plasmodium* protein families.

In common with the protozoon parasite *Trichomonas vaginalis* (Lal et al., 2005) and the free-living *Tetrahymena thermophyla* (Bright et al., 2010), *Entamoebae* encode a very large number of Rab GTPases. These genes control vesicular trafficking in the cell and the size of the gene family points to the importance and complexity of these processes in *Entamoeba*. In *E. histolytica*, 102 Rab GTPases, forming over 16 subfamilies, have been annotated (Saito-Nakano et al., 2005; Nakada-Tsukui et al., 2010). A comparison of the Rab GTPases of *E. histolytica* and *E. invadens* showed a general pattern of conservation of orthologous genes between the two species (Nakada-Tsukui et al., 2010; see Fig. 4b). This indicates that the expansion of the gene family largely occurred prior to the divergence of the two species, and suggests that complex Rab GTPase-controlled vesicular trafficking is an important feature of the genus and its machinery is conserved.

Important differences are seen between species’ gene family repertoires. Families encoding heavy- and light-chain subunits of the virulence factor Gal/GalNAc lectin occur in multiple *Entamoeba* species, but the Gal/GalNAc lectin intermediate-chain subunit genes have not been detected in species other than *E. histolytica* and *E. dispar* (Clark et al., 2007). The cysteine protease family occurs in both *E. histolytica* and *E. dispar* (Bruchhaus et al., 1996), but the key virulence factor cysteine protease-5 is a pseudogene in *E. dispar* (Willhoeft et al., 1999b). Southern blot evidence indicates that the ariel surface proteins in *E. histolytica* are not present, or are highly divergent, in *E. dispar* (Willhoeft et al., 1999a).

## 6 Genetic diversity and population structure within *E. histolytica*

The *E. histolytica* genome does not appear to contain microsatellites. Therefore, measurement of genetic diversity and estimation of population structures has relied upon other genetic markers, among them genes containing polymorphic internal repeat regions such as that encoding the serine-rich *Entamoeba Histolytica/Dispar* Protein (SREHP/SREDP) (Ayeh-Kumi et al., 2001; Haghighi et al., 2002, 2003, 2008; Simonishvili et al., 2005; Rivera et al., 2006; Samie et al., 2008) and chitinase (Haghighi et al., 2002, 2003). The tRNA-STR loci of *E. histolytica* have proved to be useful population genetic markers (Zaki and Clark, 2001; Zaki et al., 2003; Ali et al., 2005, 2007; Escueta-de Cadiz et al., 2010) and have been used to identify genotypes associated with different clinical manifestations (Ali et al., 2007; Escueta-de Cadiz et al., 2010). Studies of genetic diversity based upon variation in repetitive DNA (tRNA-STR, SREHP, chitinase) often indicate very high levels of diversity circulating in populations of *E. histolytica* (Ayeh-Kumi et al., 2001; Haghighi et al., 2002; Zaki et al., 2003; Ali et al., 2007; Samie et al., 2008; Escueta-de Cadiz et al., 2010) and *E. dispar* (Haghighi et al., 2008; Mojarad et al., 2009).

**Fig. 5:**
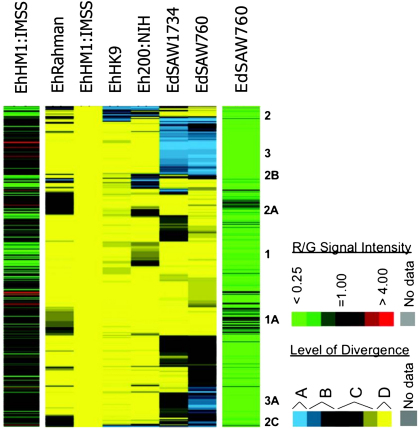
Genome-scale genetic diversity in *E. histolytica* and divergence from *E. dispar*, from Shah et al. (2005). The array was made from an *Eh*HM1:IMSS clone library. Relative hybridisation to the array was colour-coded to represent divergence: A = blue = absent or highly divergent; B = significantly divergent; C = moderately divergent; D = yellow = highly conserved; grey = missing data (see Shah et al., 2005 for definitions). The genomic abundance for each clone is represented by a green to red scale (green for low genomic abundance, R/G < 0.25, red for high genomic abundance, R/G > 4.0) in the leftmost and in the rightmost columns, for *E. histolytica* (HM1:IMSS) and *E. dispar* (SAW760), respectively. Labels to the right of the figure indicate patterns of variation: 1 and 1A = divergent in one *Eh* strain; 2, 2A, 2B, and 2C = divergent in *Eh* and *Ed* strains; 3 and 3A = divergent in both *Ed* strains but conserved in all *Eh* strains.

In contrast, studies of single nucleotide polymorphism suggest more limited diversity. Comparative genomic hybridisation studies of *E. histolytica* and *E. dispar* strains (Fig. 5) suggest that genome-wide diversity among *E. histolytica* strains is rather low (Shah et al., 2005). Sequence analysis of defined regions supports this (Bhattacharya et al., 2005; Ghosh et al., 2000). Such low single nucleotide diversity suggests a relatively recent common ancestor for *E. histolytica*. This is not inconsistent with a more rapid mutation mechanism leading to diverse repetitive regions. Such a model of population history has been proposed for *E. histolytica* (Ghosh et al., 2000).

Patterns of polymorphism often reflect population structures. In Japan, diversity among parasites infecting men who have sex with men is high, while diversity is much more limited among parasites infecting residents of institutions (Haghighi et al., 2002). Similar low diversity among parasites infecting residents of institutions was seen in the Philippines, where clear population structuring was observed within and between locations (Rivera et al., 2006). In South Africa, genotypes clustered within households but showed extensive diversity among different households (Zaki et al., 2003). Repetitive DNA markers appear to be stable enough to link closely related parasites, recently transmitted among members of a household, an institution, or recent sexual partners (Salit et al., 2009). However, the variability of these markers, as indicated by the extremely extensive diversity they show in endemic populations, suggests that larger-scale, longer-term population structure may be undetectable using them, and that SNP markers may be preferable in some situations.

An important unanswered question about the population structure of *Entamoeba* is whether they are predominantly sexual or clonal. The *E. histolytica* genome project revealed a complement of genes necessary for meiosis, pointing to the possibility of sex in natural populations (Ramesh et al., 2005; Logsdon, 2008; Loftus et al., 2005; Stanley, 2005). This is significant because sexual populations can exchange genes such as virulence and drug resistance genes, generating selectively advantageous genotypes that can spread rapidly through populations. Genetic exchange has been demonstrated in other species of protozoa previously believed to be clonal. *Giardia lamblia* (Poxleitner et al., 2008; Cooper et al., 2007), *Leishmania major* (Akopyants et al., 2009), *Trypanosoma brucei* (Gibson et al., 2008) and *Trypanosoma cruzi* (Gaunt et al., 2003) all show evidence of sex. Determining patterns of linkage disequilibrium between genetic markers in endemic *E. histolytica* populations (specifically, does linkage disequilibrium decrease with increasing distance between genetic markers as in obligately sexual protozoa such as *P. falciparum* (Conway et al., 1999)?) should allow us to answer the question definitively.

## 7 Concluding remarks

Many questions remain concerning the evolution of *Entamoeba* species, related to the complex architecture of the genome and to the structure of *Entamoeba* populations. The ability to rapidly generate whole genome sequences may help to answer some of these questions, both by allowing comparative analyses of genomes to be undertaken within and between species and by identifying genetic markers for use in molecular epidemiological studies. The challenge is to translate these new data into more effective interventions against the disease.

## Figures and Tables

**Table 1:** Statistics describing sequenced *Entamoeba* genomes.^a^

	*E. histolytica*	*E. dispar*	*E. invadens*
Num. of scaffolds	1496	3312	1149
Num. of megabases	20.80	22.96	40.89
N50^b^	49118	27840	243235
Coverage^c^	8x	4.32x	2.8x
Num. of genes	8333	8748	11549

a Numbers represent data in AmoebaDB version 1.1.

b 50% of bases are in scaffolds of this size or greater.

c As stated in genome project information in GenBank.
